# Weakly Acidic Bile Is a Risk Factor for Hypopharyngeal Carcinogenesis Evidenced by DNA Damage, Antiapoptotic Function, and Premalignant Dysplastic Lesions In Vivo

**DOI:** 10.3390/cancers13040852

**Published:** 2021-02-18

**Authors:** Clarence T. Sasaki, Sotirios G. Doukas, Panagiotis G. Doukas, Dimitra P. Vageli

**Affiliations:** The Yale Larynx Laboratory, Department of Surgery (Otolaryngology), Yale School of Medicine, New Haven, CT 06510, USA; clarence.sasaki@yale.edu (C.T.S.); sotirios.doukas.mail@gmail.com (S.G.D.); panagiotis.doukas@yale.edu (P.G.D.)

**Keywords:** in vivo, bile, weakly acidic reflux, laryngopharyngeal reflux, NF-*κ*B, DNA damage, hypopharyngeal cancer, head and neck cancer

## Abstract

**Simple Summary:**

The etiologic role of biliary reflux in hypopharyngeal cancer is supported by clinical data. Although, reflux episodes often occur at pH 4.0, they can also occur at weakly acidic pH (5.5–6.0). The carcinogenic effect of bile at strongly acidic pH (pH 3.0) was recently documented in vivo. Here, we provide novel in vivo evidence that a weakly acidic pH of 5.5, similarly to a strongly acidic pH of 3.0, increases the risk of bile-related hypopharyngeal neoplasia. We document that chronic exposure of hypopharyngeal mucosa to bile at pH 5.5 promotes premalignant lesions with DNA damage, NF-*κ*B activation, and deregulated mRNA and miRNA phenotypes, including *Bcl-2* and *miR*-451a. The oncogenic effects of bile over a wider pH range suggests that antacid therapy may be insufficient to fully modify the effects of a bile induced oncogenic effect.

**Abstract:**

Background: There is recent in vivo discovery documenting the carcinogenic effect of bile at strongly acidic pH 3.0 in hypopharynx, while in vitro data demonstrate that weakly acidic bile (pH 5.5) has a similar oncogenic effect. Because esophageal refluxate often occurs at pH > 4.0, here we aim to determine whether weakly acidic bile is also carcinogenic in vivo. Methods: Using 32 wild-type mice C57B16J, we performed topical application of conjugated primary bile acids with or without unconjugated secondary bile acid, deoxycholic acid (DCA), at pH 5.5 and controls, to hypopharyngeal mucosa (HM) twice per day, for 15 weeks. Results: Chronic exposure of HM to weakly acidic bile, promotes premalignant lesions with microinvasion, preceded by significant DNA/RNA oxidative damage, γH2AX (double strand breaks), NF-*κ*B and p53 expression, overexpression of *Bcl*-2, and elevated *Tnf* and *Il6* mRNAs, compared to controls. Weakly acidic bile, without DCA, upregulates the “oncomirs”, *miR*-21 and *miR*-155. The presence of DCA promotes *Egfr*, *Wnt5a*, and *Rela* overexpression, and a significant downregulation of “tumor suppressor” *miR*-451a. Conclusion: Weakly acidic pH increases the risk of bile-related hypopharyngeal neoplasia. The oncogenic properties of biliary esophageal reflux on the epithelium of the upper aerodigestive tract may not be fully modified when antacid therapy is applied. We believe that due to bile content, alternative therapeutic strategies using specific inhibitors of relevant molecular pathways or receptors may be considered in patients with refractory GERD.

## 1. Introduction

Laryngopharyngeal tumors are mainly squamous cell carcinomas and are among the most aggressive and devastating cancers. Tobacco smoking, alcohol consumption and HPV infection have historically been closely linked to laryngopharyngeal cancers. However, laryngopharyngeal reflux (LPR) has been causally implicated epidemiologically [1,2]. Almost 50–86% of GERD (gastroesophageal reflux disease) patients have been shown to present with mixed gastric and duodenal fluids in esophageal refluxate suggesting that enterogastric reflux may be much more prevalent than previously appreciated [3,4,5,6,7,8]. Others indicate that biliary esophageal refluxate frequently reaches the upper aero-digestive tract and that it may represent a causative risk factor in carcinogenesis [9].

Using an in vivo mouse model, we recently demonstrated that conjugated bile at a strongly acidic pH of 3.0 caused invasive squamous cell carcinoma in exposed hypopharyngeal mucosa (HM) [10,11,12]. We also defined the essential antiapoptotic role of NF-*κ*B as a mechanistic link in supporting its tumorigenic effect in vitro and in vivo [13,14,15,16,17,18,19] and that hypopharyngeal cancers harvested from patients with documented biliary esophageal reflux exclusively demonstrated highly activated NF-*κ*B and characteristic mRNA and miRNA phenotypes vs. controls similar to those described in our laboratory models [20].

The key role of NF-*κ*B in cancer initiation and progression, including head and neck squamous cell carcinomas (HNSCC), has been widely discussed and documented by its complex interactions with multiple other signaling molecules and pathways, including TNF/IKK/Bcl-2, EGFR/Ras/RAF/MAPK, IL-6/STAT3, Akt/PI3K/mTOR, and wnt/β-catenin [21,22,23,24,25]. NF-*κ*B can also interact with microRNA (miRNA) molecules in HNSCC [26]. Specifically, NF-*κ*B activation has been associated with the regulation of oncogenic *miR*-21 and “tumor suppressor” *miR*-34a [14,16,26,27]. In addition, downregulation of “tumor suppressor” *miR*-451a can promote cell proliferation by activating NF-*κ*B [28]. Deregulations of “oncomirs” *miR*-21, *miR*-155, and *miR*-192 or “tumor suppressor” *miR*-34a, *miR*-375 and *miR*-451a have recently been linked to supraesophageal malignancies [29,30,31,32,33].

Although patients with GERD classically experience symptoms when the pH drops to 4.0 [34], many patients with refractory GERD also show that weakly acidic refluxate at pH 5.5–6.0 induces typical GERD symptoms [35,36]. This observation suggests the possibility that bile may also be active in weakly acidic conditions and that specific bile salts, such as unconjugated secondary bile, deoxycholic acid (DCA), may play a prominent role within this pH range. Although bile found in esophageal aspirate largely consists of conjugated primary bile acids, secondary unconjugated forms such as DCA can also be identified [7,37]. Depending on the pH, bile acids can be found in both ionized and unionized forms, the latter considered more harmful due to their enhanced ability to interact with the cell membrane of epithelial cells [38]. At pH less than 3.0, bile salts tend to precipitate whereas between pH 5.5-7.0 most conjugated primary bile acids are found to be ionized and therefore relatively, although not completely, inactive. However, DCA, an unconjugated secondary bile acid, remains unionized (pKa 5.5–6.5) and therefore capable of interacting with and penetrating cell membranes of epithelial cells at a weakly acidic pH range [39]. This relationship may be specifically relevant because DCA is known to be tumorigenic in esophagus and colon as well [40,41,42].

We hypothesize that long-term exposure of laryngopharyngeal mucosa to gastroduodenal fluid (primary conjugated bile with and without unconjugated DCA) at a weakly acidic pH may promote a neoplastic process documented by histopathologic changes preceded by (i) DNA damage, (ii) acceleration of an NF-*κ*B-related oncogenic profile, and (iii) miRNA deregulations, previously linked to an acidic bile-induced oncogenic effect [12]. 

## 2. Results

### 2.1. Chronic Exposure of Hypopharynx to Bile at Weakly Acidic pH 5.5 Induced Pre-Malignant Changes with Elevated DNA Damage

To investigate whether chronic exposure of HM to weakly acidic bile can cause histopathologic alterations that support premalignant or malignant changes, we used histologic staining (hematoxylin and eosin, H&E) and performed histologic evaluation based on previously established criteria [43,44,45]. 

**Figure 1:**
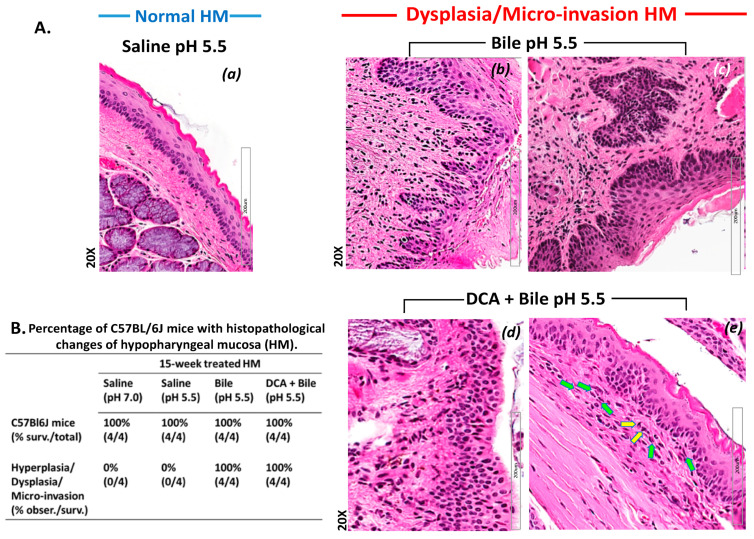
Weakly acidic bile-induced pre-malignant changes in murine hypopharyngeal squamous mucosa (HM) of C57Bl/6J mice (**A**). (H&E staining) (from left to right). *Normal HM:* (**a**) keratinized stratified squamous epithelium/single layer of basal cells; *Dysplastic HM*: (**b**) mild dysplastic epithelium with rete ridges shape; (**c**) thickness of stratified epithelium and hyperchromatic or pleiomorphic basal cells extend into the upper layers of the mucosa and well differentiated squamous epithelial cells forming islands with architectural changes in submucosa and underlying muscle with hyperchromatic nuclei. *Dysplasia-Micro-invasion:* (**d**) submucosal invasion by basal cells while HM maintaining full thickness nuclear hyperchromatism without surface maturation, and (**e**) atypical neoplastic cells in the submucosa characterized by mitotic figures (yellow arrows) and possible intercellular bridges of the atypical cells (green arrows). (**B**). Percentage (%) of C57BL/6J mice exhibiting histopathological alterations of HM after 15 weeks of exposure.

Microscopic examination of HM exposed for 15 weeks to bile at a weakly acidic pH of 5.5 revealed pre-malignant lesions, consisting of dysplasia and micro-invasion (Figure 1A(a–e)).

Specifically, HM exposed to weakly acidic bile without DCA demonstrated well differentiated squamous epithelial cells forming islands in submucosa with hyperchromatic nuclei (Figure 1A(c)). In addition, HM exposed to weakly acidic bile with DCA demonstrated dysplastic lesions and micro-invasion of neoplastic cells into submucosa (Figure 1A(d,e)).

Pre-malignant changes were detected in all weakly acidic bile-treated cases, compared to controls (100% vs. 0%), which showed normal histological patterns (Figure 1B).

### 2.2. Chronic Exposure of Bile at Weakly Acidic pH 5.5 with or without DCA on Hypopharynx Increased Cell Proliferation and DNA Damage and Decreased E-Cadherin

#### 2.2.1. Ki67 Cell Proliferation Marker and Cytokeratin

To investigate the effect of chronic weakly acidic bile exposure on enhancing cell proliferation in hypopharyngeal squamous cell epithelium, we performed immunohistochemical analysis (IHC) for Ki67 cell proliferation marker and CK14, a specific marker for basal layer keratinocytes [46]. 

**Figure 2:**
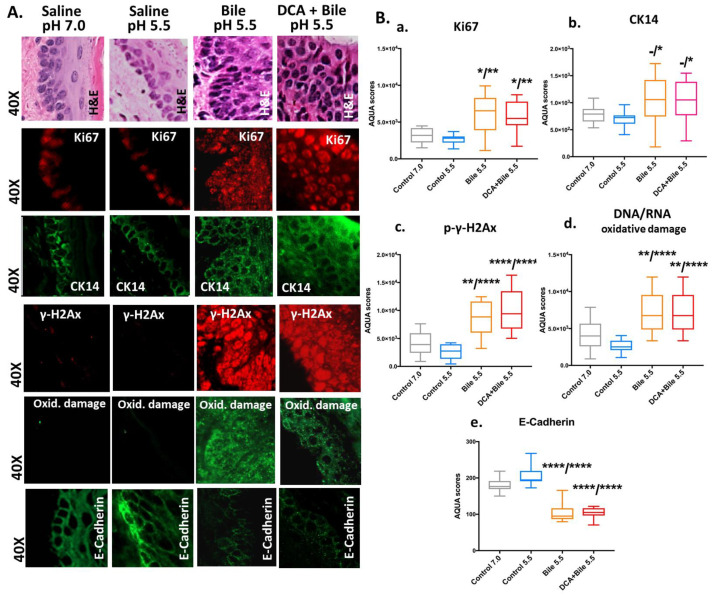
Molecular alterations underlying weakly acidic bile-induced histopathological changes of murine hypopharyngeal mucosa (HM) after 15 weeks of exposure, linked to increased cell proliferation (Ki67 and CK14), DNA damage, and decreased cell–cell interactions (E-Cadherin). **A**. Immunofluorescence staining and automated quantitative analysis (AQUA) were used for Ki67, γΗ2ΑΧ, CK14, DNA/RNA oxidative damage (Oxid. damage), and E-Cadherin [DyLight^®^549 used for red; DyLight^®^488 used for green; DAPI was used for nuclei staining (not seen here)]. **B**. Graphs created by GraphPad Prism 7.0 indicate a statistically significant difference of AQUA-score means for (**a**) Ki67 (nuclear), (**b**) CK14 (nuclear and cytoplasmic), (**c**) γΗ2ΑΧ (nuclear), (**d**) DNA/RNA oxidative damage markers (nuclear and cytoplasmic), and (**e**) E-Cadherin (membrane/cytoplasmic) between weakly acidic bile with or without DCA vs. controls (* *p* < 0.01; ** *p* < 0.001; *** *p* < 0.0001; **** *p* < 0.00001; by *t*-test; multiple comparisons by Holm-Sidak; GraphPad Prism 7.0).

As shown in Figure 2, IHC analysis revealed that HM exposed to bile at a weakly acidic pH of 5.5 with or without DCA produced an intense staining for cell proliferation markers Ki67 (red) and CK14 (green), expanding in parabasal or suprabasal layers, compared to controls that showed a less intense staining limited to the basal layer (Figure 2A; Supplementary material, Figure S1A).

Using AQUA, scoring for Ki67 and CK14 revealed significantly higher expression levels of these epithelial cell proliferation markers in bile treated-HM compared to controls (Figure 2B(a,b)) (*p* < 0.05, *t*-test; means±SD; multiple comparisons by Holm-Sidak). 

#### 2.2.2. DNA Oxidative Damage and γH2Ax, a Marker for DBSs

To explore if chronic weakly acidic bile exposure can cause DNA oxidative damage and double-strand breaks (DSBs) in murine HM, we performed IHC analysis using DNA/RNA oxidative damage markers (oxo8dG, 8-hydroxy-20 -deoxyguanosine; oxo8Gua, 8-oxo-7,8-dihydroguanine; and oxo8G, 8-oxo-7,8-dihydroguanosine), and γH2Ax, a specific marker for DSBs [47].

In Figure 2, IHC analysis revealed that HM exposed to bile at a weakly acidic pH of 5.5 with or without DCA produced intense staining for γH2Ax (red) and DNA/RNA oxidative damage (green) markers, expanding in parabasal or suprabasal layers, compared to controls that showed weak or absent staining (Figure 2A; Supplementary material, Figure S1B).

Using AQUA, scoring for γH2Ax and DNA/RNA oxidative damage markers (Figure 2B(c,d)) revealed significantly higher expression levels of these markers in bile treated-HM compared to controls (*p* < 0.005, *t*-test; mean ± SD; multiple comparisons by Holm-Sidak). 

The above observations suggest that at weakly acidic pH 5.5 a portion of primary bile acids, which may remain un-ionized (and therefore active) in combination with unionized secondary bile acid, DCA, at pH > 5.0, serve as potent activators of an anti-apoptotic process and capable of inducing a profound DNA damage in long-term topically exposed HM. 

#### 2.2.3. Cell Adhesion Molecule E-Cadherin

To detect whether chronic weakly acidic bile exposure can alter cell–cell interactions in murine HM, we performed IHC analysis for E-Cadherin, the cell adhesion molecule [48,49]. 

IHC analysis revealed that HM exposed to bile at a weakly acidic pH of 5.5 with or without DCA produced a less intense staining for E-Cadherin staining (green), compared to controls demonstrating an intense E-cadherin staining within its entire thickness (Figure 2A), suggesting that long-term exposure of HM to bile at weakly acidic pH induces changes in cell–cell interactions. Using AQUA, scoring for E-Cadherin revealed significantly lower expression levels in bile treated-HM compared to controls (Figure 2B(e)) (*p* < 0.00005, *t*-test; means ± SD; multiple comparisons by Holm-Sidak).

### 2.3. Chronic Exposure of Bile at Weakly Acidic pH 5.5 with or without DCA on Hypopharynx Increased NF-κB Activation and p53 Levels

#### 2.3.1. NF-κB (p65 S536)

In order to document that chronic weakly acidic bile exposure can activate NF-*κ*B in murine HM, we performed IHC analysis for p-NF-*κ*B (p65 S536) and analyzed its nuclear levels, compared to controls.

**Figure 3:**
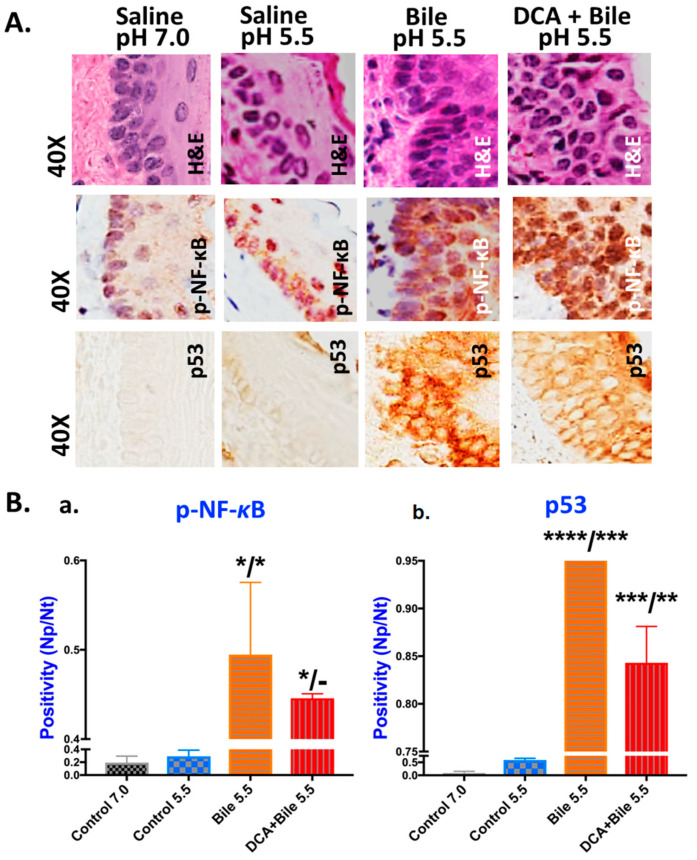
Weakly acidic bile-induced NF-*κ*B activation and increased expression of p53 in murine hypopharyngeal mucosa (HM) after 15 weeks of exposure. (**A**). Immunohistochemical analysis (IHC), using chromogenic staining, was performed for p-NF-*κ*B (p65 S536) (brown) and p53 (brown). (**B**). Graphs created by GraphPad Prism 7.0 indicate a statistically significant difference of (**a**) nuclear positivity for p-NF-*κ*B and (**b**) cytoplasmic positivity for p53 (by Image Scope software) between weakly acidic bile with or without DCA vs. controls (* *p* < 0.01; ** *p* < 0.001; *** *p* < 0.0001; **** *p* < 0.00001; by *t*-test; multiple comparisons by Holm-Sidak; GraphPad Prism 7.0).

Chromogenic staining for p-NF-*κ*B (p65 S536) of murine HM exposed to bile with or without DCA at weakly acidic pH 5.5 revealed abundant NF-*κ*B activation, as evidenced by intense p-NF-*κ*B nuclear staining of cells in basal and suprabasal layers, particularly at sites of pre-malignant lesions (Figure 3A). Controls showed less intense and less extensive NF-*κ*B activation in HM, limited to the basal layer only (Figure 3A).

Chronic exposure of HM to bile at a weakly acidic pH induced significantly higher levels of nuclear p-NF-*κ*B (p65 S536) compared to controls, supporting an enhanced NF-*κ*B activation, as shown in Figure 3B(a) (*p* < 0.05, *t*-test; means ± SD; multiple comparisons by Holm-Sidak). 

#### 2.3.2. p53 

To detect if chronic weakly acidic bile exposure can enhance p53 expression in murine HM, we performed IHC analysis for p53 (detecting either mutant or wild form) and analyzed its expression levels relative to controls. 

Chromogenic staining for p53 of murine HM exposed to bile at a weakly acidic pH with or without DCA revealed intense p53 staining, particularly at sites of pre-malignant lesions. Controls presented absence or weak staining of p53 (Figure 3A). 

Chronic exposure of HM to bile at a weakly acidic pH induced significantly higher levels of p53 compared to saline-treated controls, as shown in Figure 3B(b) (*p* < 0.005, *t*-test; means ± SD; multiple comparisons by Holm-Sidak). 

### 2.4. Correlation among Cell Proliferation Markers, Cell-Cell Adhesion Molecules, DNA Damage Markers, p-NF-κB and p53 

In order to estimate the correlation coefficient between expression levels of the analyzed markers in the studied groups, we performed Spearman non-parametric correlations. 

Spearman analysis revealed a significant inverse correlation between Ki67 and E-Cadherin protein levels (*r* = −0.9956, *p* = 0.0022, one-tailed) and a strong positive correlation between Ki67 and CK14 protein levels (*r* = 0.9925, *p* = 0.0038; one-tailed).

Spearman analysis also revealed a strong positive correlation between p-NF-*κ*B and p53 positivity (*r* = 1, *p* = 0.0417; two-tailed) and between γH2AX and CK14 (*r* = 1, *p* = 0.0417; two-tailed), suggesting that NF-*κ*B and p53 activation, as well as DNA damage and cytokeratin overexpression, consisted of strong relationships that are altered in parallel, under the effect of bile at weakly acidic pH. 

### 2.5. Chronic Exposure of HM to Primary Bile Acids at Weakly Acidic pH 5.5 with or without DCA Induced Elevated mRNA Levels of Anti-Apoptotic Bcl-2 and Cancer Related Cytokines

To explore whether chronic exposure of murine HM to weakly acidic bile can activate anti-apoptotic mRNA phenotype, we performed gene expression analysis, by reverse transcription and quantitative PCR (qPCR) analysis, for a panel of NF-*κ*B related oncogenic factors previously linked to acidic bile-induced hypopharyngeal carcinogenesis [10,12,13,14,15,16,17,18,19]. 

**Figure 4:**
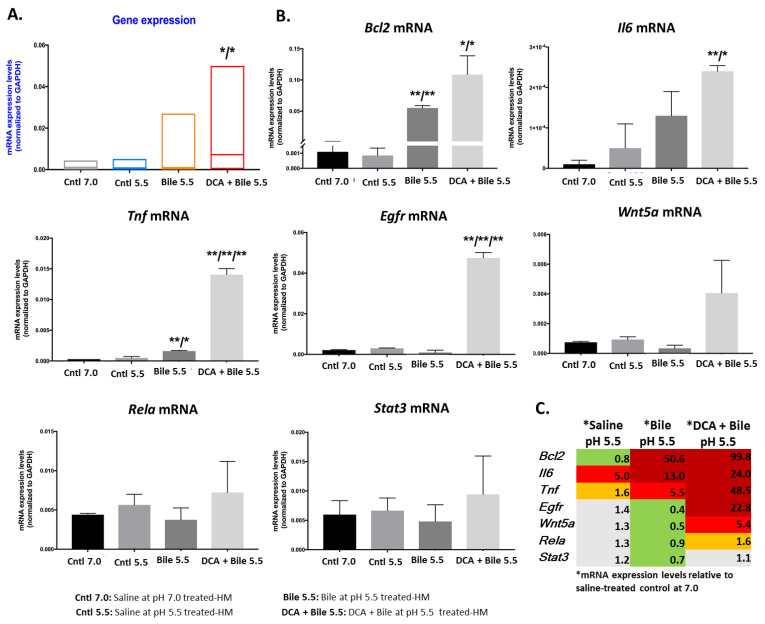
Weakly acidic bile-induced transcriptional activation of NF-*κ*B-related oncogenic phenotype in murine hypopharyngeal mucosa (HM) after 15 weeks of exposure. (**A**) Graph depicts mRNA levels of all analyzed NF-*κ*B-related genes (*Bcl2*, *Il6*, *Tnf*, *Egfr*, *Wnt5a*, *Rela*, and *Stat3*) after exposure to bile at pH 5.5 with or without DCA vs. controls (*p* value using one-way ANOVA, Friedman test; Graph Pad Prism software 7.0). (**B**) Graphs indicate transcriptional levels of each analyzed gene in HM exposed to weakly acidic bile vs. controls (* *p* < 0.01; ** *p* < 0.001; *t*-test; multiple comparisons using Holm-Sidak; GraphPad Prism 7.0). (**C**) Table shows the changes of the mRNA oncogenic phenotype (fold-change of mRNAs) caused by bile with or without DCA at weakly acidic pH 5.5 vs. control (saline at pH 7.0). (By real-time qPCR analysis; data obtained from four analyzed samples).

Our analysis revealed that murine HM exposed to weakly acidic bile with DCA expressed the highest transcriptional levels of the analyzed oncogenic factors relative to controls (*p* < 0.05, by Friedman, Dunn’s multiple comparisons test) (Figure 4A; Supplementary material, Table S1).

Specifically, chronic exposure of HM to bile (conjugated primary bile acids) with or without unconjugated secondary bile acid, DCA, at pH 5.5, induced significantly higher mRNA levels of anti-apoptotic *Bcl2* and cancer-related inflammatory *Tnf* compared to HM exposed to controls (Figure 4B) (*p* < 0.00005; *t*-test multiple comparisons using Holm Sidak method).

Furthermore, exposure of murine HM to primary bile acids with DCA (pH 5.5) induced a significant overexpression of *Egfr* and *Il6*, and an upregulation of *Wnt5a* and *Rela* compared to controls (Figure 4B,C).

Spearman non-parametric test revealed statistically significant positive correlation between transcriptional levels of (i) *Tnf* and *Il6*, (ii) *Egfr* and *Wnt5a*, (iii) Egfr and *Rela*, and (iv) *Rela* and *Wnt5a* (*r* = 1, *p* = 0.042; two-tailed).

### 2.6. Chronic Exposure of HM to a Mixture of Primary Bile Acids at Weakly Acidic pH 5.5 Induced an Upregulation of Inflammatory and Cancer Related miR-21 and miR-155 and Downregulation of “Tumor Suppressor” miR-375 and miR-451a.

To investigate if chronic exposure of murine HM to weakly acidic bile can deregulate cancer-related miRNA phenotype, we performed miRNA analysis, by reverse transcription and qPCR, for a panel of NF-*κ*B related “oncomirs” and “tumor suppressor” miRNAs, previously linked to acidic bile-induced hypopharyngeal carcinogenesis [11,12,14,16,17,18]. 

**Figure 5:**
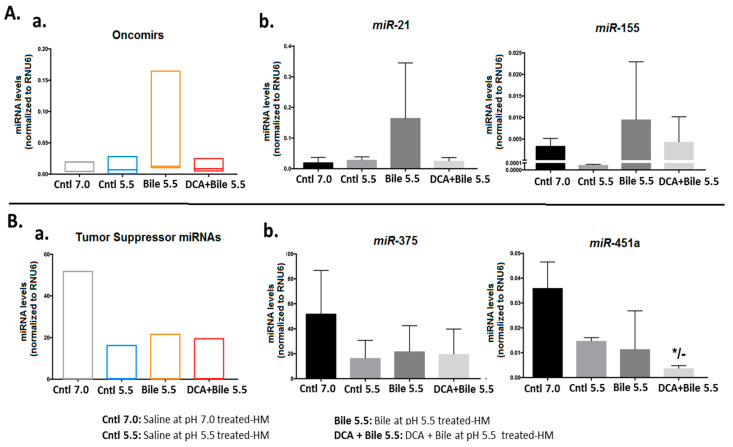
Weakly acidic bile-induced deregulation of the miRNA oncogenic phenotype in murine hypopharyngeal mucosa (HM) after 15 weeks of exposure. (**A**) (**a**) Graph depicts total “oncomirs” levels after exposure to bile at pH 5.5 with or without DCA vs. controls (one-way ANOVA; Graph Pad Prism software 7.0) (**b**) Graphs indicate expression levels for each analyzed “oncomir” *miR*-21 and *miR*-155 after exposure to weakly acidic bile vs. controls (Graph Pad Prism software 7.0). (**B**) (**a**) Graph depicts total “tumor suppressor” miRNA levels after exposure to bile at pH 5.5 with or without DCA vs. controls (one-way ANOVA; Graph Pad Prism software 7.0) (**b**). Graphs indicate expression levels for each analyzed “tumor suppressor” *miR*-375 and *miR*-451a after exposure to weakly acidic bile vs. controls. (* *p* < 0.01; *t*-test; multiple comparisons using Holm-Sidak; GraphPad Prism 7.0). (By real-time qPCR analysis; data obtained from four analyzed samples).

Micro-RNA analysis revealed that chronic exposure of HM to primary bile acids at a weakly acidic pH of 5.5 induced an upregulation of “oncomirs” (Figure 5A(a); Supplementary Table S2), particularly of *miR*-21 and *miR*-155 (Figure 5A(b)). 

We also observed that chronic exposure of HM to weakly acidic environment induced a downregulation of “tumor suppressor” miRNAs (Figure 5Ba; Supplementary material, Table S2). Chronic exposure of murine HM to bile with DCA at weakly acidic pH (5.5) induced significantly lower levels of “tumor suppressor” *miR*-451a compared to controls (*p* < 0.05) (Figure 5B(b)). 

Spearman non-parametric test revealed a significant inverse correlation between *miR*-451a and *Tnf* mRNAs (*r* = –1, *p* = 0.042; two-tailed). On the other hand, a statistically significant positive correlation was found between expression levels of *miR*-155 and *Stat3* mRNAs (*r* = 1, *p* = 0.042; one-tailed).

## 3. Discussion

A brief explanation may help to place the origin of DCA into clinical perspective. Most bile salts secreted through the common bile duct into the duodenum are reabsorbed downstream by intestinal mucosa and transported through the portal circulation back to the liver allowing them to be re-secreted thus describing a continuous enterohepatic re-circulation. DCA, a secondary product derived from primary cholic acid by the action of intestinal microbial enzymes, ultimately finds its way into hepatic secretions by this enterohepatic mechanism. Therefore, the mixed composition of secreted bile consisting of primary and secondary bile acids can result in a broad cumulative effect over a wider range of pH values. 

We have previously showed that DCA alone can produce a significant activation of NF-*κ*B and promote preneoplastic lesions in murine laryngopharyngeal mucosa [10,11]. Here, we used a mixture of conjugated primary bile acids with DCA to mimic “physiologic” refluxate content that has been found in aspirates from GERD patients. Our selection of conjugated primary bile acids at 10 mmol/L is based on clinical observations that bile acids can approach a concentration of >10 mmol/L in esophageal refluxate [1,19]. Similarly, we used DCA at a concentration of 0.28 mmol/L, based on values previously described in GERD patients [7,37].

The role of NF-*κ*B as a key factor in bile-related esophageal carcinogenesis has been previously reported [42,50,51,52]. Although, the mechanism by which NF-*κ*B may mediates bile tumorigenic effect in squamous cell carcinoma of the upper aerodigestive tract is not well explained, we previously documented the central role of NF-*κ*B in acidic bile-related progressive mutagenic effect in hypopharyngeal mucosa [10,11,12]. We proved that acidic bile-induced early neoplastic events can be successfully inhibited by both pharmacologic and dietary NF-*κ*B inhibitors, further understanding the antiapoptotic role of NF-*κ*B in this process [13,14,15,16,17,18,19]. 

Here, we document that the chronic local effect of weakly acidic bile at pH 5.5 can induce NF-*κ*B activation and molecular alterations in premalignant murine HM, similarly to bile at a strongly acidic pH 3.0 [10,11,12]. We demonstrate that weakly acidic bile is very capable of promoting transcriptional activation of NF-*κ*B associated genes participating in oncogenic pathways in HNSCC, including inflammatory molecules, such as *Tnf* and *Il6*, antiapoptotic *Bcl-2*, and oncogenic factors *Egfr*, *Wnt5a,* and *Rela* [21,22,23,24,25]. We also show that weakly acidic bile can promote the deregulation of miRNAs, small regulatory RNA molecules have been previously linked to HNSCC, such as “oncomirs” *miR*-21 and *miR*-155 [26,30], as well as “tumor suppressor” *miR*-451a, [32]. The “oncomirs” *miR-*21 and *miR-*155 have been suggested as important markers of poor prognosis in head and neck cancer [23,53,54,55], and may be directly regulated by NF-*κ*B [56,57,58,59]. In addition, *miR-*451a has been previously linked to hypopharyngeal carcinogenesis and shown to affect NF-*κ*B activation [32,33]. These data further support the mechanistic role of NF-*κ*B in bile-related hypopharyngeal carcinogenesis and provide new evidence that enterogastric refluxate at weakly acidic pH can constitutively activate NF-*κ*B and its related antiapoptotic signaling, supporting an underlying tumorigenic process. 

We further provide evidence that the presence of DCA in bile refluxate accelerates the upregulation of NF-*κ*B related oncogenic factors (Figure 4A) and induce premalignant squamous epithelial lesions and microinvasion in murine hypopharynx. These data support that bile consisting of DCA at a weakly acidic pH of 5.5 can interact with the hypopharyngeal epithelial cells and thus exert its carcinogenic potency on the upper aerodigestive tract. We also demonstrate that weakly acidic bile without DCA can promote the upregulation of NF-*κ*B related “oncomirs” (Figure 5A), and cause premalignant lesions, implying that partially active conjugated primary bile acids at a weakly acidic pH of 5.5 can stimulate an oncogenic process. 

We show that weakly acidic bile-induced dysplastic lesions of murine HM, present an abundant DNA/RNA oxidative damage and DSBs, elevated epithelial cells proliferation rates, and altered cell-cell interactions that are linked to tumor-initiating mutations and support a microinvasion progression [46,48,49,60,61]. 

The role of tumor suppressor gene *Trp53*, is also known to regulate the cell cycle and its overexpression may contribute to tumorigenesis [62]. So, although in normal tissue p53 is expressed at low levels, its abundant expression (of mutant or wild p53 form) may have a role in the neoplastic process [63]. Here, we show that weakly acidic bile-induced premalignant lesions produce an elevated overall p53 protein expression relative to controls, supporting p53 contribution in weakly acidic bile-reflux neoplastic process in hypopharynx.

Identifying and characterizing weakly acidic bile fluid as an independent risk factor in head and neck malignancies would be considered novel. Such recognition would allow for more comprehensive risk stratification and prevention of laryngopharyngeal cancers. We believe that the identification of specific molecular pathways or receptors activated by bile in the upper aerodigestive tract, similar to those have previously identified in gastrointestinal tract and lower esophagus, would allow therapeutic intervention in patients with bile reflux, especially those experience symptoms after treatment with antacid therapy. 

## 4. Materials and Methods

### 4.1. In Vivo Model 

We used *Mus Musculus*, wild-type mice C57BL/6J (Jax mice, Jackson Laboratory USA). Our study included 16 males and 16 females (8 mice per group; 4 males + 4 females). We topically treated murine HM, two times per day for 15 weeks with experimental or control fluids. 

Bile treatment included a mixture of conjugated primary bile acids (10 mmol/L in buffered saline) (~4 µmol per day) with and without unconjugated secondary bile acid deoxycholic acid (0.28 mmol/L) (~0.1 µmol per day) (DCA; Alfa Aesar^®^, Tewksbury, MA, USA) at concentrations previously described in GERD patients [5,7,37], at a weakly acidic pH of 5.5, adjusted with 1 M HCl. 

The experimental and control groups included (i) bile at pH 5.5, (ii) bile with DCA at pH 5.5, (iii) saline at pH 5.5 (weakly acidic control), and (iv) saline at pH 7.0 (reference control for feeding tube mechanical effect on HM). Procedures were performed in parallel with at least 6-h intervention between the treatments, followed the approved protocol 2020-11039, by IACUC; Yale University. 

At the end of 15 weeks and 30 min after the last treatment, the animals were euthanized using CO_2_ according to IACUC euthanasia policy and guidelines (updated American Veterinary Medical Association (AVMA) Guidelines for the Euthanasia of Animals: 2020 Edition) and HM tissue was dissected from all animals. Four HM tissue specimens of two males and two females coming from each group were put into 10% neutral buffered formalin (Thermo Fisher Scientific, Middletown, VA, USA) and then embedded to paraffin (Yale Pathology Facilities). The remaining four HM tissue specimens were immersed in RNA stabilization solution (RNAlater, Life Technologies, Grand Island, NY, USA) and stored at −80 °C for molecular analysis.

### 4.2. Histologic Evaluation

We used hematoxylin and eosin (H&E) staining in 3 µm thick tissue sections of formalin fixed and paraffin embedded HM. We examined at least two H&E stained tissue sections from each HM specimen (two males and two females) coming from each experimental and control group, by light microscopy, based on previously established criteria [43,44] and laboratory mouse histology [45]. Images were captured and analyzed by Aperio CS2, Image Scope software (Leica microsystems, Buffalo Grove, IL, USA), as previously described [10,11,12].

### 4.3. Immunohistochemical (IHC) Analysis

We used chromogenic and immunofluorescence staining in at least two tissue specimens from each experimental (including those with premalignant/malignant lesions) and control group, to detect (i) molecular changes of NF-*κ*B (p65 pSer536), tumor suppressor p53, cell proliferation markers Ki67 and CK14, and cell–cell adhesion molecule E-Cadherin, as well as (ii) DNA damage, using γH2AX, a marker of DNA double-strand breaks (DSBs) and oxidative DNA/RNA damage markers, related to bile at weakly acidic pH, as previously described [10,11,12]. Specifically, we used anti-DNA/RNA oxidative damage antibody (Anti-DNA/RNA Damage antibody, clone 15A3, Abcam) with a high specificity and affinity to oxo8dG (8-hydroxy-2′-deoxyguanosine), oxo8Gua (8-oxo-7,8-dihydroguanine), and oxo8G (8-oxo-7,8-dihydroguanosine), to detect oxidative DNA damage in dysplastic layers and the foci of invasion. 

Positive controls and non-template negative control, were used in each IHC assay, as recommended by the manufacturer. Microscopic examination after chromogenic staining was performed using a Leica light microscope and were captured and analyzed by Image Scope software and Aperio CS2, respectively (Leica Microsystems, Buffalo Grove, IL, USA). Nuclear p-NF-*κ*B was expressed as a ratio of positive nuclei to total number of nuclei (defined as p-NF-*κ*B positivity), while total p53 protein levels were expressed as total positive to total number ratios (defined as p53 positivity). For fluorescence staining we used anti-rabbit or anti-mouse secondary DyLight^®^488 (green), DyLight^®^549 (red) for target proteins and DAPI (blue) to distinguish positive nuclei (DyLight^®^488 and DyLight^®^549; Vector Labs, USA). Microscopic examination and image analysis after immunoflurescense staining was performed using Zeiss fluorescence microscope and AxionVision system (Carl Zeiss microscopy, White Plains, NY, USA).

Data from two independent images per tissue section (at least four tissue sections per group) were included for statistical analysis (means ± SD; by multiple *t*-test).

### 4.4. Gene Expression and miRNA Analysis

We used qPCR (CFX96^TM^, Bio-Rad) to analyze mRNA levels of target genes (normalized to *Gapdh* reference control) (Supplementary material, Table S3) and specific miRNAs (normalized to *RNU6* reference control) (Supplementary material, Table S4), as previously described [10,11,14]. Briefly, total RNA was isolated (RNeasy mini kit; Qiagen^®^, Germantown, MD, USA) from four specimens (two males and two females) of each experimental and control group, and its concentration and quality were determined by absorption at 260 nm, and ratios at 260/280 nm (>2.0), respectively (NanoDrop^TM^ 1000 spectrophotometer; Thermo Scientific). 

To analyze mRNA levels, we performed reverse transcription to cDNA (Whole Transcriptome kit; Qiagen^®^, Germantown, MD, USA), followed by qPCR analysis, using specific primers for mouse genome (QuantiTect^®^ primers assay, Qiagen^®^, Germantown, MD, USA) (Supplementary Table S3).

To analyze miRNA levels, we used total RNA and miScript II RT kit (Qiagen, Louisville, KY) and performed reverse transcription synthesis, followed by qPCR analysis, using specific primers for mouse genome (miScript Primer Assays; Qiagen^®^, Germantown, MD, USA) (Supplementary Table S4). 

Relative mRNA or miRNA expression levels were estimated for each target gene or miRNA relative to the reference controls (*Gapdh* or *RNU6*) (ΔΔCt).

### 4.5. Statistical Analysis 

We performed ONE-WAY ANOVA (Friedman or Kruskal–Wallis; Dunn’s multiple analysis test; *p* values < 0.05) and *t*-test (multiple comparisons by Holm-Sidak), using GraphPad Prism 7.0 software, to reveal evidence of statistically significant changes of DNA damage markers, Ki67, CK14, E-Cadherin, NF-*κ*B, p53, gene expression (mRNA), or miRNA levels between experimental and control groups. Spearman non-parametric correlations were used to estimate the correlation coefficient between expression levels of the analyzed markers in the studied groups (*p* values < 0.05). 

## 5. Conclusions

We provide in vivo evidence that bile at a weakly acidic pH, similar to bile at a strongly acidic pH, is capable of inducing DNA damage, NF-*κ*B-mediated antiapoptotic function, and histologic changes consistent with a premalignant phenotype. This new evidence suggests that the oncogenic properties of biliary esophageal reflux on the epithelium of the upper aerodigestive tract may be not fully modified when antacid therapy is applied. We believe that due to bile content, alternative therapeutic strategies may be considered in patients with refractory GERD. Future research protocols, including specific inhibitors of relevant molecular pathways or bile receptors on the epithelium of the upper aerodigestive tract, may increasingly play a more prominent role in containment strategies.

## Data Availability

Data is contained within the article or supplementary material.

## Supplementary Materials

The following are available online at https://www.mdpi.com/2072-6694/13/4/852/s1, Figure S1: Weakly acidic bile treated murine hypopharyngeal mucosa and squamous epithelial cells formatted islands in submucosa with increased proliferative rates and possible DNA double-strand breaks (DSBs); Table S1: (A) The 15-week weakly acidic bile-induced transcriptional levels of NF-κB-related oncogenic pathway in murine hypopharyngeal mucosa. (B) Relative mRNA expression ratios for each target gene in murine hypopharyngeal mucosa exposed to weakly acidic bile for 15 weeks relative to weakly acidic control; Table S2: (A) The 15-week weakly acidic bile-induced miRNA levels in exposed murine hypopharyngeal mucosa. (B) Relative miRNA expression ratios for each marker in murine hypopharyngeal mucosa exposed to weakly acidic bile for 15 weeks relative to weakly acidic control; Table S3: Mouse genes (target and reference *Gapdh*) and their detected transcripts, analyzed by real time qPCR, in murine hypopharyngeal mucosa; and Table S4: Mouse mature miRNAs (targets) and reference RNU6-2 small RNA control, analyzed using real-time qPCR, in murine hypopharyngeal mucosa.
