# Supplementary material for: Weakly Acidic Bile Is a Risk Factor for Hypopharyngeal Carcinogenesis Evidenced by DNA Damage, Antiapoptotic Function, and Premalignant Dysplastic Lesions In Vivo
Source: Cancers (Basel). 2021 Feb 18;13(4):852. doi: 10.3390/cancers13040852 (PMC7923205; doi:10.3390/cancers13040852)
Supplement: Supplementary file 1 [file cancers-13-00852-s001.pdf]

Weakly Acidic Bile Is a Risk Factor for Hypopharyngeal Carcinogenesis Evidenced by DNA Damage, Antiapoptotic Function, and Premalignant Dysplastic Lesions In Vivo

Clarence T. Sasaki †, Sotirios G. Doukas, Panagiotis G. Doukas and Dimitra P. Vageli †,*

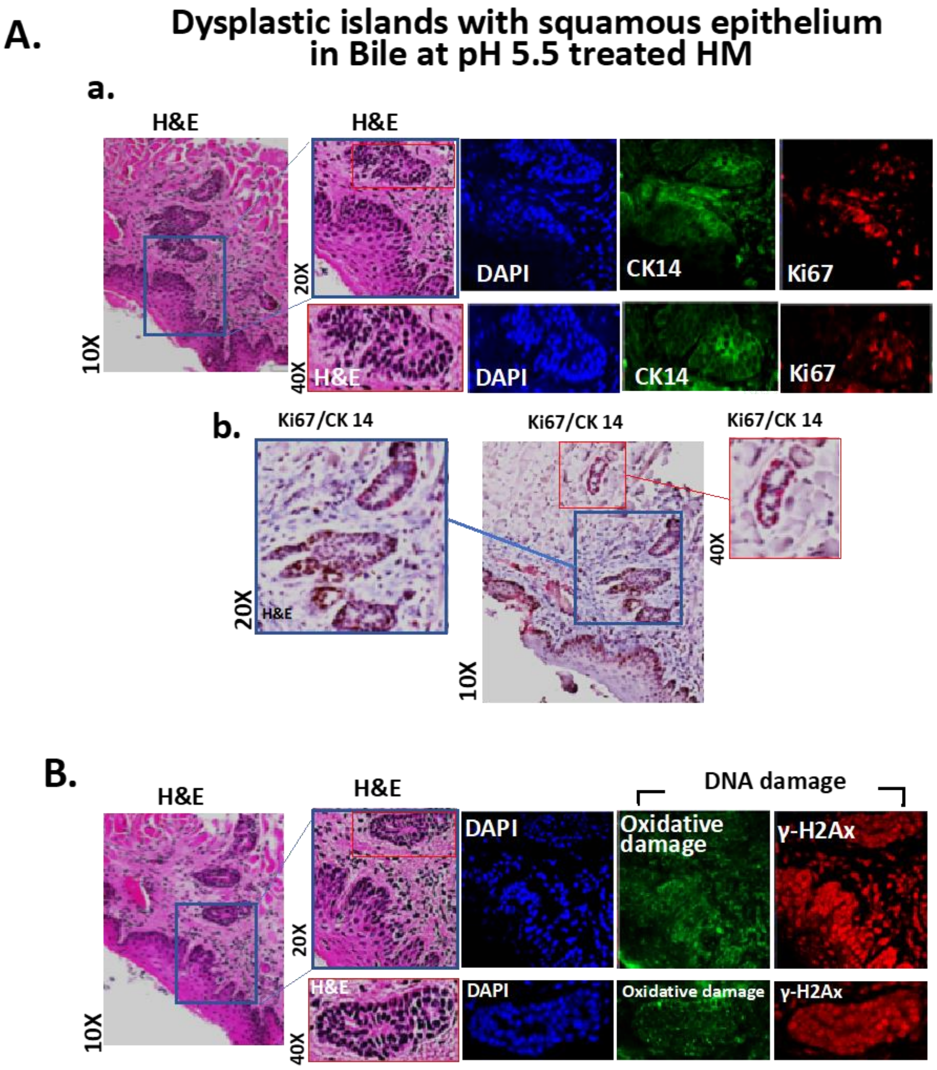

Figure S1. Weakly acidic bile treated murine hypopharyngeal mucosa (HM) and squamous epithelial cells formatted islands in submucosa with increased proliferative rates and possible DNA double strand breaks (DSBs). **A**. Immunohistochemical analysis (IHC) for cytokeratin (CK14) and cell proliferation marker Ki67 using, (a) fluorescence staining (CK14: green; Ki67: red; DAPI for nucleus: blue) and (b) chromogenic staining (CK14: pink; Ki67: dark red). **B**. IHC analysis for DNA/RNA oxidative damage markers and γ-H2Ax(S139), using fluorescence staining (DNA/RNA damage: green; γ-H2AX: red; DAPI for nucleus: blue).

Table S1. A. The 15-week weakly acidic bile-induced transcriptional levels of NF-κB related oncogenic pathway in murine hypopharyngeal mucosa (HM).

	15-weeks treated-HM			
	**Saline pH 7.0**	**Saline pH 5.5**	**Bile pH 5.5**	**DCA+Bile pH 5.5**
Bcl2	1.09E-03	8.50E-04	5.51E-02	1.09E-01
Il6	1.00E-05	5.00E-05	1.30E-04	2.40E-04
Tnf	2.90E-04	4.70E-04	1.61E-03	1.41E-02
Egfr	2.08E-03	3.00E-03	9.10E-04	4.75E-02
Wnt5a	7.48E-04	9.35E-04	3.40E-04	4.06E-03
Rela	4.40E-03	5.64E-03	3.75E-03	7.22E-03
Stat3	4.27E-03	5.14E-03	2.80E-03	4.80E-03

B. Relative mRNA expression ratios for each target gene in murine hypopharyngeal mucosa exposed to weakly acidic bile for 15 weeks relative to weakly acidic control.

**Target gene/*Gapdh* (*ΔΔ*^{CT})**	**Bile 5.5/ Saline 5.5**	**DCA+Bile 5.5/ Saline 5.5**
Bcl2	1.97E+00	4.71E-01
Il6	1.85E+00	8.04E+00
Tnf	8.76E+00	2.13E+01
Egfr	5.22E+01	9.39E+01
Wnt5a	1.19E+01	2.88E+00
Rela	1.93E+00	1.68E+01
Stat3	1.71E+00	3.22E+01

Table S2. A. The 15-week weakly acidic bile-induced miRNA levels in exposed murine hypopharyngeal mucosa (HM).

**Target miRNA/*RNU6** (*ΔΔ*^{CT})**	**15-weeks treated-HM**			
	**Saline pH 7.0**	**Saline pH 5.5**	**Bile pH 5.5**	**DCA+Bile pH 5.5**
miR-21	2.04E-02	2.91E-02	1.65E-01	2.58E-02
miR-155	3.42E-03	7.00E-05	9.54E-03	4.34E-03
miR-192	1.93E-02	6.96E-03	1.27E-02	8.81E-03
miR-34a5	2.04E-02	4.04E-02	8.70E-02	3.27E-02
miR-375	5.20E+01	1.65E+01	2.19E+01	1.97E+01
miR-451a	3.60E-02	1.48E-02	1.14E-02	3.71E-03

*normalization of miRNA levels using small RNA [snRNA RNU6B (RNU6-2)].

B. Relative miRNA expression ratios for each marker in murine hypopharyngeal mucosa exposed to weakly acidic bile for 15 weeks relative to weakly acidic control.

**Target miRNA/*RNU6* (*ΔΔ*^{CT})**	**Bile 5.5/ Saline 5.5**	**DCA+Bile 5.5/ Saline 5.5**
miR-21	5.70E+00	8.87E-01
miR-155	1.36E+02	6.20E+01
miR-192	1.83E+00	1.27E+00
miR-375	2.16E+00	8.09E-01
miR-34a	1.33E+00	1.20E+00
miR-451a	7.74E-01	2.52E-01

Table S3. Mouse genes (target and reference *Gapdh*) and their detected transcripts, analyzed by real time qPCR, in murine hypopharyngeal mucosa.

Gene (mouse)	Detected transcripts	Amplicon length (bp)
Gapdh	NM_008084	144
	NM_001289726	
Rela	NM_009045	82
Stat3	NM_011486	99
	NM_213659	
	NM_213660	
Wnt5a	NM_001256224	130
	NM_009524	
Bcl2	NM_009741	80
Tnf	NM_013693	112
	NM_001278601	
Egfr	NM_007912	68
	NM_207655	
Il6	NM_031168	128

Table S4. Mouse mature miRNAs (targets) and reference *RNU6-2* small RNA control, analyzed by real time qPCR, in murine hypopharyngeal mucosa.

miRNA (mouse)	Target mature miRNA, Sanger Accession)
miR-21a	mmu-miR-21a-5p, MI0000569
miR-155	mmu-miR-155-5p, MI0000177
miR-192	mmu-miR-192-5p, MI0000551
miR-375	mmu-miR-375-3p, MI0000792
miR-34a	mmu-miR-34a-5p, MI0000584
miR-451a	mmu-miR-451a, MI0001730
**Small RNA**	**Control**
RNU6	U6 small nuclear RNA, ENSMUSG00000095132
